# Atmospheric pressure neutral reionization mass spectrometry for structural analysis†
†Electronic supplementary information (ESI) available: Details for experimental procedures and additional supporting data. See DOI: 10.1039/c7sc01999h



**DOI:** 10.1039/c7sc01999h

**Published:** 2017-07-21

**Authors:** Pengyuan Liu, Pengyi Zhao, R. Graham Cooks, Hao Chen

**Affiliations:** a Center for Intelligent Chemical Instrumentation , Department of Chemistry and Biochemistry , Edison Biotechnology Institute , Ohio University , Athens , OH , USA 45701 . Email: chenh2@ohio.edu; b Department of Chemistry , Purdue University , 560 Oval Drive , West Lafayette , IN , USA 47907 . Email: cooks@purdue.edu

## Abstract

Ion dissociation is the usual basis for tandem MS analysis but a significant limitation is that only charged fragments from ion dissociation events are detected while neutral fragments are simply lost. This study reports our continued effort to solve this problem by developing atmospheric pressure neutral reionization mass spectrometry (APNR). In APNR, analyte ions are thermally dissociated (atmospheric pressure thermal dissociation, APTD) followed by soft reionization using electrosonic spray ionization (ESSI). Our results show that APNR is a powerful method for structural analysis of various biomolecules such as peptides, saccharides and nucleotides, as well as for elucidating unimolecular ion dissociation mechanisms. It was found that APNR provides extensive fragment ions including a series of y ions in peptides, which benefit sequencing and provide complementary information to collision induced dissociation (CID). In particular, direct cleavage of disulfide bonds of peptides occurs during APTD, facilitating peptide sequencing and disulfide bond mapping. In addition, many cross-ring cleavage fragments are detected during APNR analysis of oligosaccharides, indicating that the APTD dissociation process is energetic and potentially useful for identifying glycan linkage sites. Fragmentation patterns of oligosaccharide isomers can be used for their differentiation. Furthermore, in the cases of dissociation of nucleotides and synthetic naphthoylindole drugs, the putative neutral, phosphorylated riboses and indoles, were successfully detected using APNR, providing strong evidence to confirm previously proposed unimolecular ion dissociation mechanisms. We believe this APNR technique along with APTD should be of high value in structure determination of biomolecules.

## Abstract 2

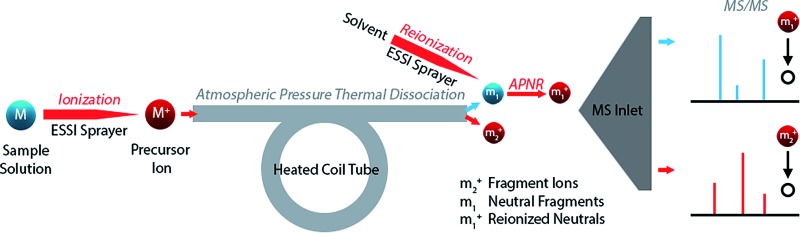
Neutral fragments from an ion dissociation event could be detected *via* reionization at atmospheric pressure.

## Introduction

Besides measuring molecular weights (MWs), another important goal of mass spectrometric analysis is to elucidate molecular structure *via* gas-phase ion dissociation. The fragment ions resulting from ion dissociation provide information about analyte structure. The greater the variety of fragment ions observed from a dissociation event, the more structural information can be gained. Although many useful dissociation techniques have been developed,^1–12^ an important limitation is that only fragment ions are detected by mass spectrometry (MS) whereas neutral fragments generated from the dissociation event are simply lost. In many situations, identification of neutral fragments is as important as that of charged fragments in improving understanding of precursor ion structures and dissociation mechanisms.

**Scheme 1:**
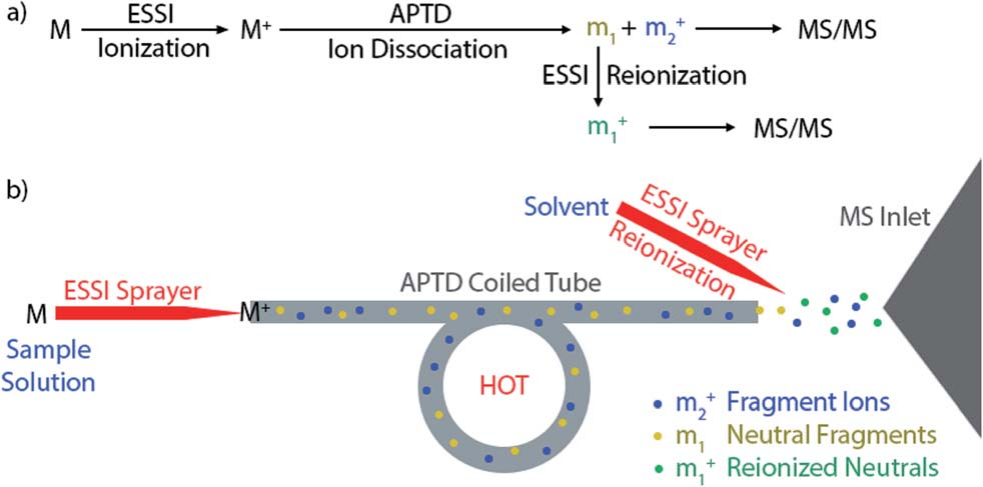
Schematic showing the processes and apparatus used for APNR-MS.

Researchers began to pay attention to the ‘lost’ neutral fragments since several decades ago. Analysis of neutral species by mass spectrometric techniques became possible when neutralization-reionization mass spectrometry (NRMS) was introduced,^13,14^ based on neutralization of a mass-selected ion beam by electron transfer collision with a gas (*e.g.*, Xe, Hg, NH_3_) in a vacuum chamber and reionization of the resulting neutrals by collision with another target (*e.g.*, O_2_) *via* electron transfer or charge stripping. Adopting the concept and technique of NRMS, neutral fragment reionization (N_f_R)^15^ was developed, in which the selected ion beam underwent collisional dissociation first. Then the charged fragments were deflected and the neutral fragments were subject to mass analysis after collisional reionization. Although N_f_R-MS provides a unique and elegant way to analyze the neutral fragments,^16–19^ this technique requires a specialized instrument and it is implemented in the mass analyzer in vacuum, which limits its utility. Furthermore, collisional reionization may lead to unwanted extensive fragmentation, obscuring the structural analysis of the target neutral fragment. For simplicity of instrumentation and soft reionization, an atmospheric pressure reionization technique would be a better choice. In this study, atmospheric pressure neutral reionization (APNR) is achieved by adding an electrosonic spray ionization (ESSI, a variant form of electrospray ionization)^20^ sprayer to reionize the neutral fragment species resulting from atmospheric pressure thermal dissociation (APTD), an ion dissociation method that we developed previously (Scheme 1).^8,21–23^ As shown in Scheme 1a and b, the first ESSI spray ionizes a target molecule M and the resulting ion M^+^ undergoes thermal dissociation in a coiled and heated tube outside the mass spectrometer to produce fragments m_1_ and m_2_^+^. A second ESSI sprayer generates charge droplets which pick up and gently ionize the neutral fragments m_1_ converting them to m_1_^+^, and making them detectable by MS. Both m_1_^+^ and m_2_^+^ could be further dissociated using traditional tandem MS analysis (*e.g.*, CID) for increased structural information (Scheme 1a). The ESSI reionization used here is expected to be softer than corona discharge which we used previously for the same purpose.^8^ It is worth noting that the concept of ionization of gas phase neutrals has been widely used in many atmospheric pressure ionization techniques.^24–27^

In this study, different types of compounds have been chosen to test the current APNR method, including peptides, saccharides, nucleotides, and synthetic drugs. Except for peptides, none of the other types of compounds has been studied before using APTD or APNR. Generally, APNR provides more fragment ions than APTD alone as neutral fragment species are ionized. In particular, oligosaccharide isomers can be distinguished by APNR. Nucleotides can be fragmented by APTD in the positive ion mode and analyzed in the negative ion mode by ESSI neutral reionization. The neutral indole fragments from synthetic naphthoylindole drugs, which are not observable in traditional CID MS/MS analysis, can be detected by APNR. In addition, the disulfide bonds in peptides are observed to undergo either elimination or reduction in APTD and subsequent MS/MS analysis can provide information for peptide sequencing and disulfide bond mapping.

## Results and discussion

### Peptides

**Fig. 1:**
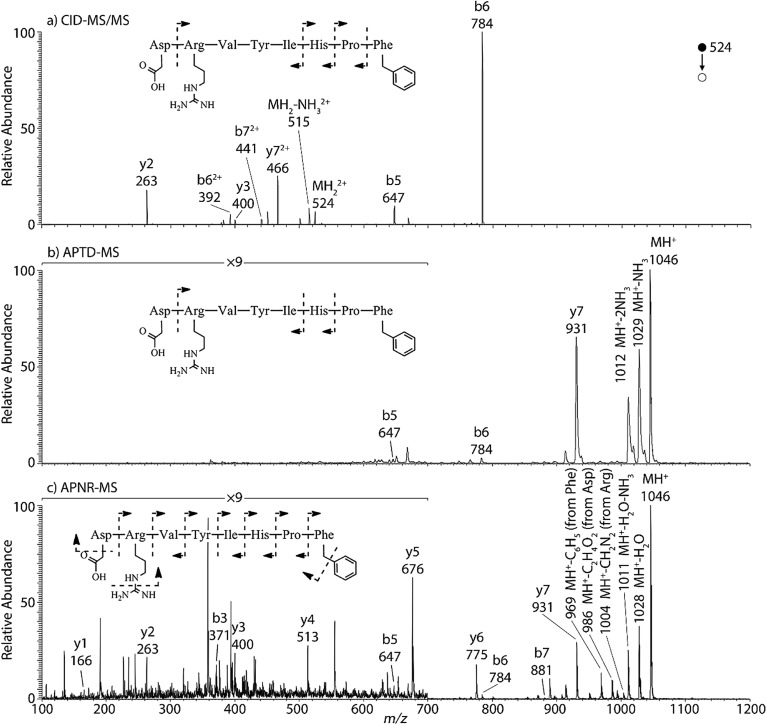
(a) CID MS/MS of +2 ion of angiotensin II (*m*/*z* 524), (b) APTD-MS and (c) APNR-MS spectra of angiotensin II.

Peptides and proteins have been widely investigated by tandem MS analysis.^28^ In CID MS/MS of peptide ions, b and y fragment ions are formed as a result of cleavage of peptide bonds. Similarly, APTD of peptide ions produces b and y fragment ions,^8^ as exemplified by data for two peptides, human angiotensin II (Fig. 1) and Gly–His–Gly (Fig. 1S†). In this study both APTD and APNR of peptides were examined and the mass spectra recorded for APNR showed more fragment ions than did APTD, including a series of y ions. Fig. 1a shows the CID MS/MS spectrum of the doubly charged angiotensin II ion (*m*/*z* 524). In the spectrum, a limited number of b and y fragments were observed, including b_5_, b_6_, b_6_^2+^, b_7_^2+^, y_2_, y_3_, and y_7_^2+^. Fig. 1b shows the APTD-MS spectrum of angiotensin II. Similarly, some b and y fragments were observed in the APTD-MS spectrum, including b_5_, b_6_, and y_7_ as well as ions from ammonia loss from the protonated angiotensin II. But the sequence coverage is still highly incomplete. Specifically, the fragment ions of y_7_ and b_6_ are dominant in the spectra since the amide cleavages N-terminal to proline (to form b_6_)^29^ and C-terminal to aspartic acid (to form y_7_)^30^ are preferred. In comparison, the APNR-MS spectrum (Fig. 1c) shows more fragments than both the CID-MS/MS and APTD-MS spectra. Significantly, in Fig. 1c, a complete series of y ions as well as b_3_, b_5_, b_6_, and b_7_ ions are observed, providing complete sequence information. In addition, fragment ions with amino acid side chain losses are also observed, including the loss of methanediimine from the arginine residue, the loss of acetic acid from the aspartic acid residue, and the loss of phenyl group from the phenylalanine residue. The assigned peaks are confirmed by CID MS/MS experiments (results are shown in Table 1S†). The formation of the complete series of y ions shown in Fig. 1c should stem from the ESSI-reionization of the corresponding neutrals generated from the peptide APTD process. Likewise, y_1_ and y_2_ were observed from the APNR spectrum of tripeptide Gly–His–Gly (Fig. 1Sb, ESI†). The distinctive series of y ions offer very useful information for peptide sequencing.

**Fig. 2:**
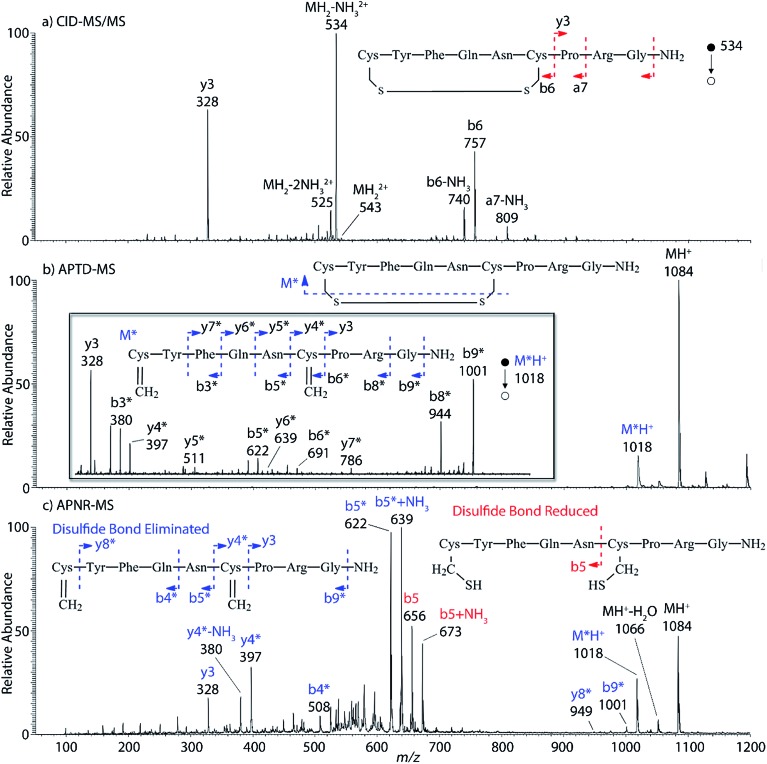
(a) CID MS/MS spectrum of +1 [Arg^8^]-vasopressin ion (*m*/*z* 534), (b) APTD-MS and (c) APNR-MS spectra of [Arg^8^]-vasopressin.

Interestingly, we also noted that disulfide bonds of peptides could be eliminated or reduced in the APTD process. This observation makes APTD different from CID, as disulfide bond cleavage is uncommon in CID in the positive ion mode. An intra-peptide disulfide bond-carrying peptide, [Arg^8^]-vasopressin (sequence is shown in Fig. 2a), was chosen for APTD and APNR experiments. Such a peptide is difficult to analyze directly CID, due to the disulfide bond constraint. Indeed, in the CID MS/MS spectrum (Fig. 2a), only a few backbone cleavage fragments, y_3_, b_6_, b_6_-NH_3_ and a_7_-NH_3_, were observed and they provided no sequence information for the backbone region covered by the disulfide bond. The APTD-MS spectrum of [Arg^8^]-vasopressin (Fig. 2b) showed no backbone fragments, except a fragment ion observed at *m*/*z* 1018, due to the elimination of the disulfide bond bridge linking Cys^1^ and Cys^6^ (labeled as M*, structure shown in Fig. 2b inset). CID MS/MS spectrum of the first generation fragment *m*/*z* 1018 (M*H^+^, Fig. 2b inset) showed many more characteristic fragment ions than did the intact peptide ion, including 
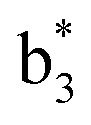
, 
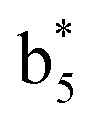
, 
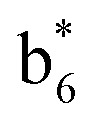
, 
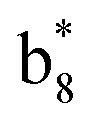
, 
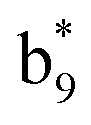
, 
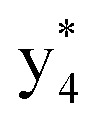
, 
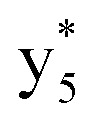
, 
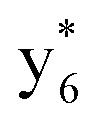
, and 
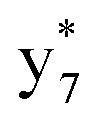
 (the superscript * indicates that the fragment ion contains modified cysteine residues due to the elimination of disulfide bond). The detection of these fragment ions not only provides increased sequence information, but also locates the 6^th^ cysteine residue in the disulfide bond, based on the presence of 
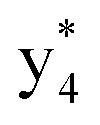
 and y_3_ fragment pairs. In addition, when APNR was applied, many fragments were directly observed in the MS spectrum without performing an addition step of CID MS/MS. These fragments (Fig. 2c) included 
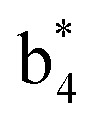
, 
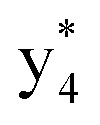
, 
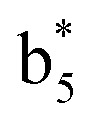
, 
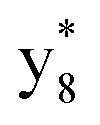
, 
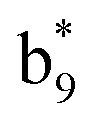
 and M*H^+^ (*m*/*z* 1018). It is also worth mentioning that the observation of 
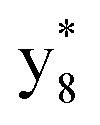
 can help to locate the 1^st^ cysteine residue. In combination with the CID MS/MS spectrum of M*H^+^ (*m*/*z* 1018, Fig. 2b inset), the exact disulfide bond location and the entire sequence of the peptide covered by disulfide bond are completely elucidated. Furthermore, interestingly, disulfide bond reduction was also observed by seeing a fully reduced b_5_ ion from the APNR-MS spectrum (Fig. 2c). All peak assignments in Fig. 2c were confirmed by further CID MS/MS analysis (results are summarized in Table 3S, ESI†). In addition, other disulfide bond-containing peptides, [Arg^8^]-vasotocin and glutathione disulfide (GSSG), were also examined and disulfide bond elimination/reduction was also observed (data is shown in the ESI†). Either reduction or elimination removes the disulfide bond constraints, converts the peptides into linear peptides and makes sequencing much easier. This result suggests great potential for APNR in proteomics research, as traditional analysis of disulfide bond-containing proteins/peptides needs chemical reduction which is time-consuming and troublesome.

### Carbohydrates

**Fig. 3:**
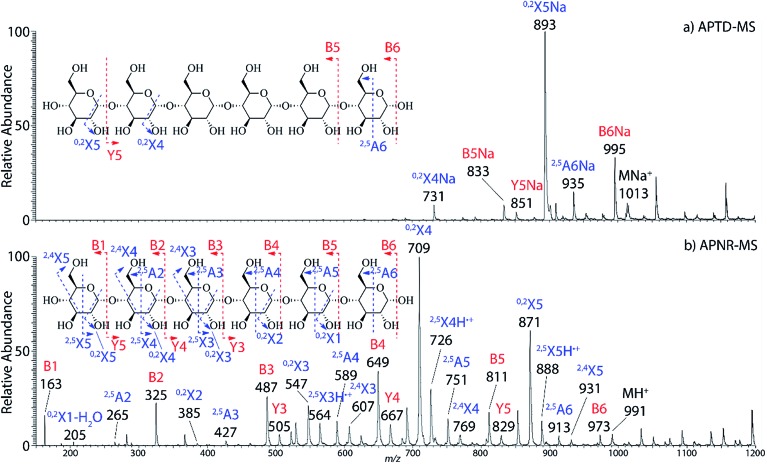
(a) APTD-MS and (b) APNR-MS spectra of maltohexaose. See Fig. 4S† for the corresponding CID MS/MS data.

**Fig. 4:**
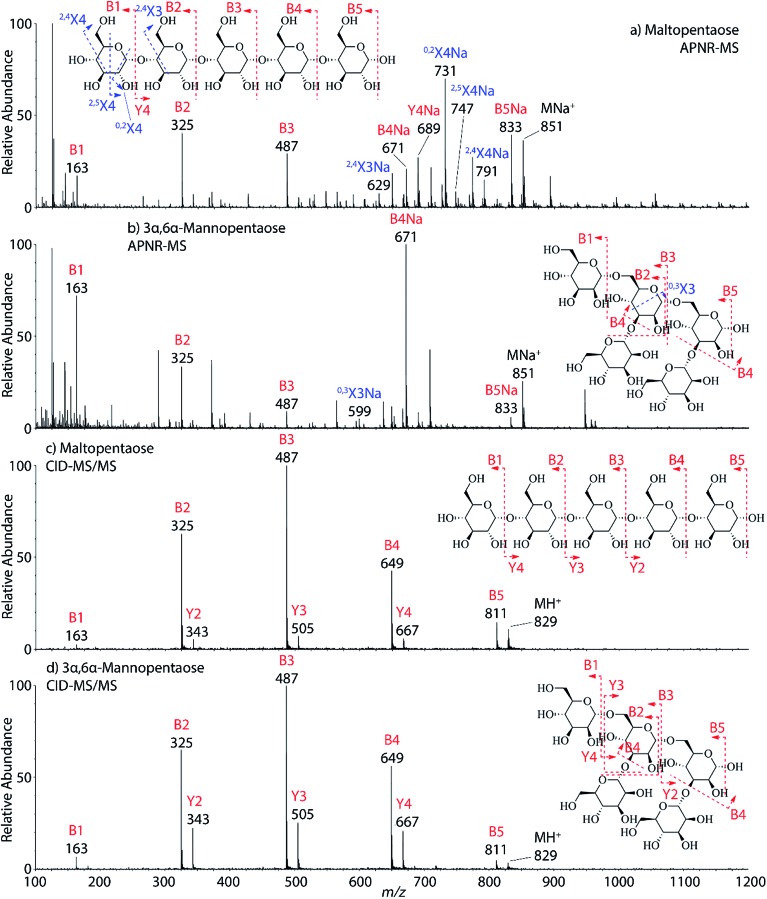
APNR-MS spectra of (a) maltopentaose and (b) 3α,6α-mannopentaose; and CID MS/MS spectra of (c) the protonated maltopentaose (*m*/*z* 829) and (d) the protonated 3α,6α-mannopentaose (*m*/*z* 829).

In addition to peptide analysis, analysis of sugars is also possible by APTD and APNR. Maltohexaose was chosen as a test example. A comparison of APTD and APNR spectra is made in Fig. 3. APTD of maltohexaose yielded only a few fragment ions including B_6_, ^2,5^A_6_, ^0,2^X_5_, Y_5_, B_5_, and ^0,2^X_4_ (seen as the sodiated ions in the spectrum). The nomenclature of saccharide fragmentation follows the system reported by Domon and Costello, in which B and Y ions represent fragments resulting from the cleavage of glycosidic bonds, and A and X ions represent fragments from the cross-ring cleavages within a sugar ring.^31^ Subscripts following the capital letters represent the number of monosaccharide units remaining in the fragment ions. In particular, for A and X ions, the two superscript numbers represent the cleaved covalent bonds. A limited number of fragment ions were observed in the high *m*/*z* range and lower mass fragment ions were completely missing in the APTD-MS spectrum (Fig. 3a). In comparison, the APNR-MS spectrum (Fig. 3b) is more informative. A full series of B ions, from B_1_ to B_6_, was observed, which covers the entire backbone of the analyte. In addition, several Y ions were observed, including Y_3_, Y_4_, and Y_5_. Besides this, extensive A and X ions from cross-ring cleavages also occurred. All the fragmentation pathways are indicated in the inset to Fig. 3b. It is worth mentioning that these A and X ions were not observed in the CID MS/MS spectrum of maltohexaose (Fig. 4S, ESI†), which only contained B and Y ions. Typically, glycosidic bond cleavage is more facile than ring cleavage, as it is observed in low energy CID MS/MS analysis.^32^ By comparison, cross-ring cleavages require more energy, indicating that APTD dissociation process is energetic. The extensive cross-ring cleavages could potentially provide very useful structural information for determining linkage sites in glycans.^33–35^ Also, APNR-MS has no mass cutoff as exemplified by observing low *m*/*z* fragments (*e.g.*, B_1_ ion) in Fig. 3b, which is another advantage compared to CID MS/MS (Fig. 4S, ESI†).

Two isomeric oligosaccharide pentamers, maltopentaose and 3α,6α-mannopentaose, were analyzed and differentiated by APNR-MS. These two isomers contain five glucose and mannose units, respectively, and their two monosaccharide units are well-known as a pair of C-2 epimers. Their backbone linkages differ slightly as well. Maltopentaose is a single chain pentamer linked by α(1→4) bonds while 3α,6α-mannopentaose contains a branched monosaccharide unit in its structure. These structural differences between the two isomers are hard to distinguish by CID MS/MS and the spectra show no differences (Fig. 4c and d). However, they are readily differentiated by APNR-MS (Fig. 4a and b). First, the APNR-MS spectrum of 3α,6α-mannopentaose shows a dominant fragment ion at *m*/*z* 671, corresponding to the sodiated B_4_ ion resulting from the cleavage of the branched monosaccharide unit. Since 3α,6α-mannopentaose contains one branch, the dominant fragment B_4_ ions might be the result of loss of the branched mono-unit. In contrast, the dominant peak observed in the APNR-MS spectrum of maltopentaose is the sodiated ^0,2^X_4_ peak (*m*/*z* 731) with a relatively low abundance sodiated B_4_ ion, which might indicate that no branched monosaccharide exists in its backbone structure. Second, the X ions observed for these two monosaccharide isomers were different as well. For maltopentaose, ^2,4^X_4_, ^2,5^X_4_, ^0,2^X_4_, and ^2,4^X_3_ ions were observed. In particular, the observation of ^0,2^X_4_ and ^2,4^X_3_ suggests the glycosidic bond is 1→4 linked without a branch unit, confirming the precursor as a maltose-like sugar. By contrast, a ^0,3^X_3_ ion was observed in the APNR-MS spectrum of 3α,6α-mannopentaose, exactly as expected for the 1→6 linked glycosidic bond and the branch 1→3 linked glycosidic bond (Fig. 4b). This example demonstrates the potential of using APNR as a powerful tool for differentiating sugar isomers.

### Nucleotides

**Fig. 5:**
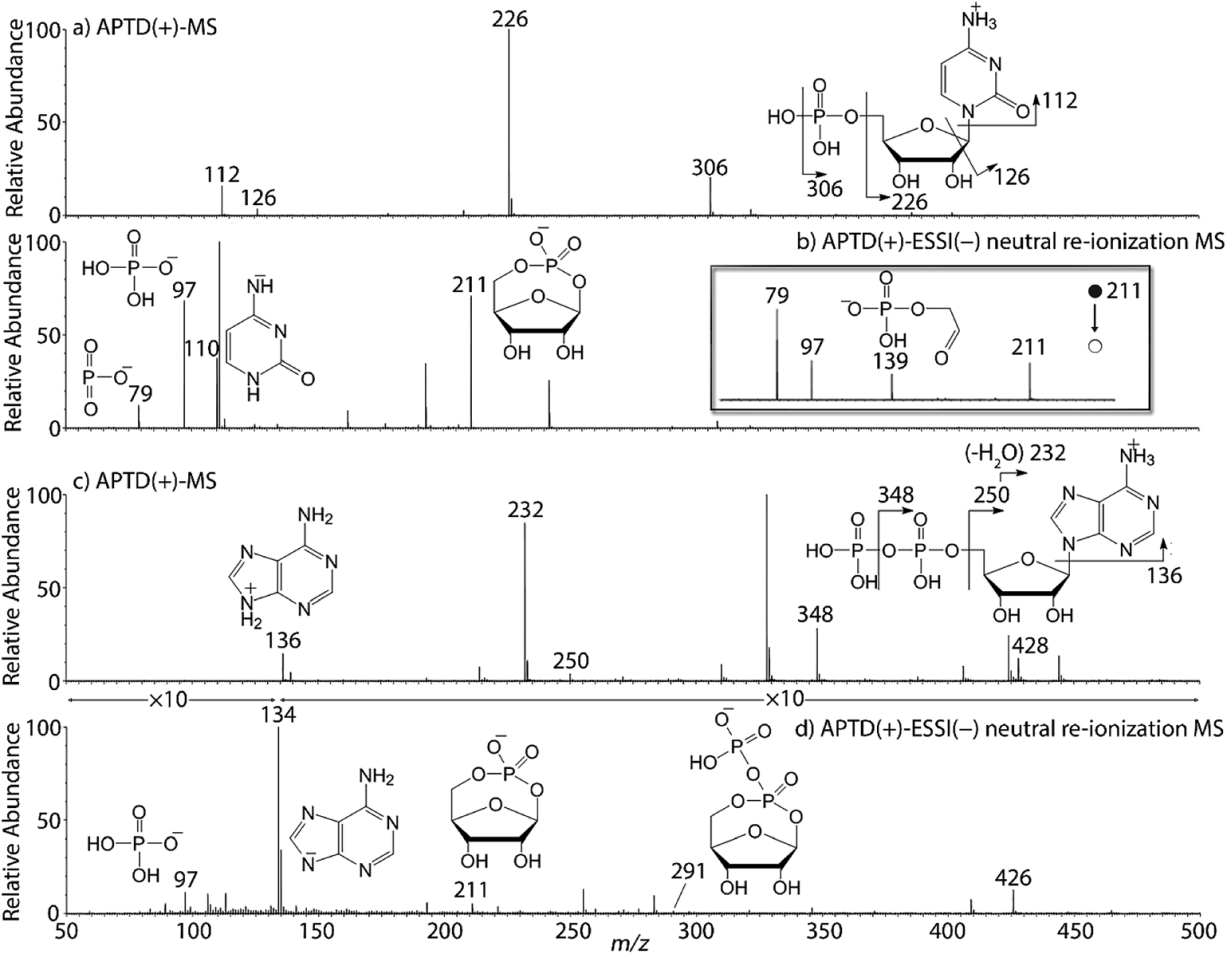
(a) APTD-MS and (b) APNR-MS spectra of CMP; (c) APTD-MS and (d) APNR-MS spectra of ADP.

**Scheme 2:**
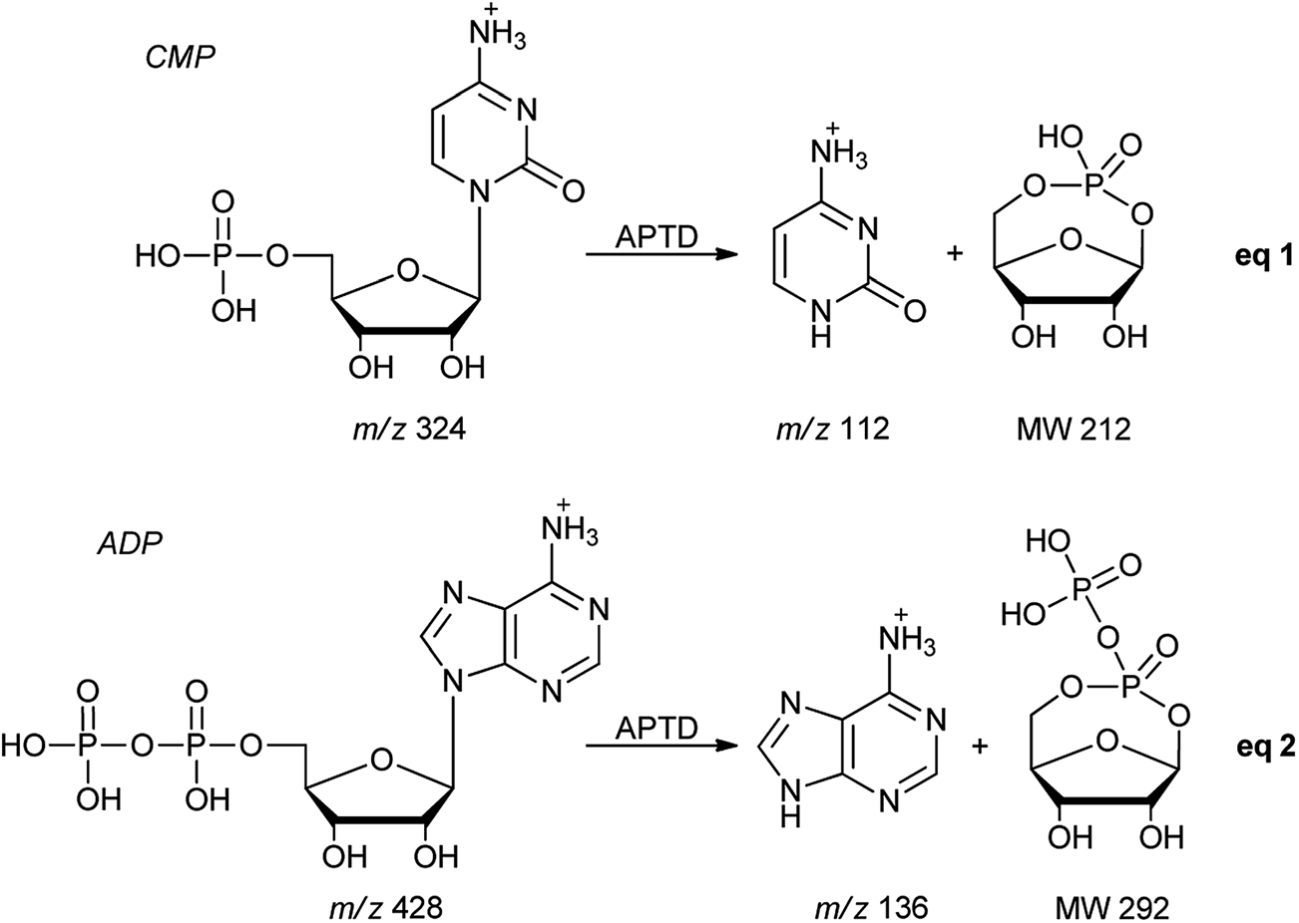
Proposed fragmentation pathways of CMP and ADP.

In addition to peptides and sugars, nucleotides are another important type of biomolecule which we examined using APTD-MS and APNR-MS. Cytidine 5′-monophosphate (CMP) was first examined and the recorded spectra are shown in Fig. 5. In the APTD-MS spectrum acquired in the positive ion mode (Fig. 5a), the ion at *m*/*z* 226 due to the loss of phosphate group was observed as the dominant peak. In addition, the protonated cytosine resulting from the loss of phosphorylated ribose was observed at *m*/*z* 112. However, the lost neutral fragments of the phosphate and phosphorylated ribose were missing from the APTD spectrum. It has been proposed that the neutral loss fragment undergoes cyclization to form a fused ring structure (eqn (1), Scheme 2).^15^ In order to investigate this hypothesis, APNR-MS of CMP was performed. In contrast to the experiments of peptides and sugars using the positive ion mode, the negative ion mode was used in the ESSI reionization step because most compounds containing phosphate groups prefer to be deprotonated in the gas phase. As shown in Fig. 5b, the negative APNR spectrum clearly displays the deprotonated phosphorylated ribose fragment at *m*/*z* 211 which corresponds to the neutral fragment proposed in eqn (1), Scheme 2. Upon CID, *m*/*z* 211 (Fig. 5b inset) dissociated into fragment ions at *m*/*z* 79, 97, and 139 by consecutive losses of C_3_H_4_O_2_, CH_2_O and H_2_O, in agreement with the ion assignment. Different from the previous N_f_R-MS study in which ionizing neutral fragments *via* collisional reionization caused extensive fragmentation,^15^ the intact neutral was observed with high intensity, which indicated again that ESSI used in this APNR experiment is a soft reionization method. In addition, phosphate and dehydrated phosphate anions were observed at *m*/*z* 97 and 79, respectively (Fig. 5b), in line with the observation that phosphate is lost during the formation of the fragment ion *m*/*z* 226 in the APTD process (Fig. 5a). Also, the deprotonated cytosine was observed at *m*/*z* 110.^36,37^

Another nucleotide, adenosine 5′-diphosphate (ADP), was also tested. Similarly, the APTD-MS spectrum in the positive ion mode shows some phosphate loss peaks at *m*/*z* 348, 250, and 232, and the protonated adenine fragment at *m*/*z* 136 (Fig. 5c). Again, the APNR-MS spectrum acquired in the negative ion mode (Fig. 5d) displays the characteristic anion of diphosphate ribose at *m*/*z* 291, a neutral species produced concomitantly with the formation of the protonated adenine at *m*/*z* 136 during the APTD process (eqn (2), Scheme 2). Other ions observed in Fig. 5d include deprotonated phosphorylated ribose at *m*/*z* 211, phosphate anion at *m*/*z* 97, and deprotonated adenine at *m*/*z* 134.

### Synthetic drugs

**Fig. 6:**
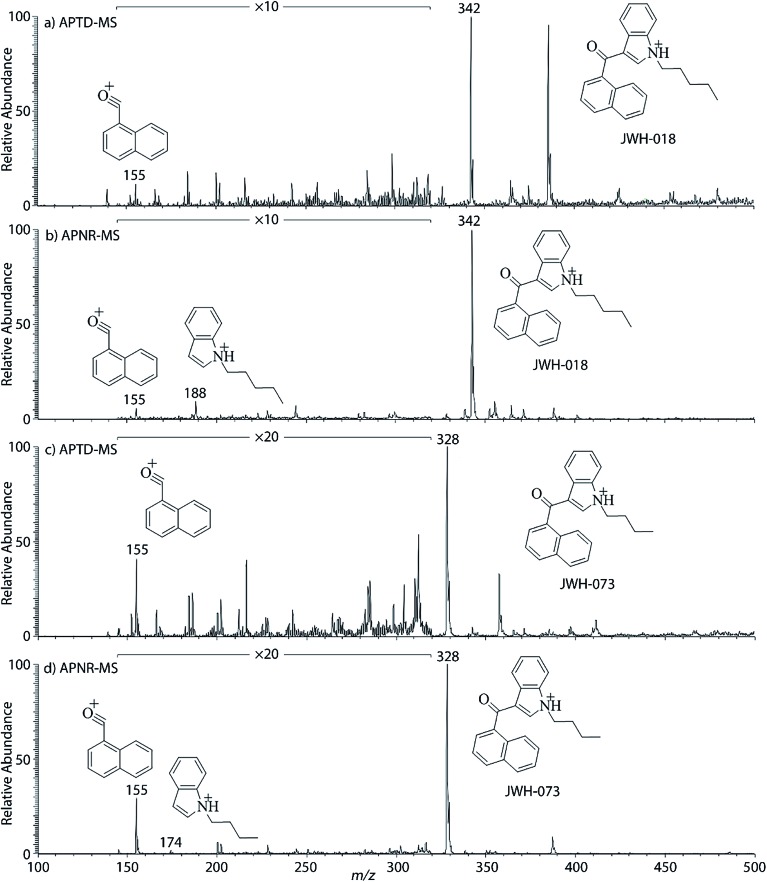
(a) APTD-MS and (b) APNR-MS spectra of JWH-018; (c) APTD-MS and (d) APNR-MS spectra of JWH-073.

**Scheme 3:**
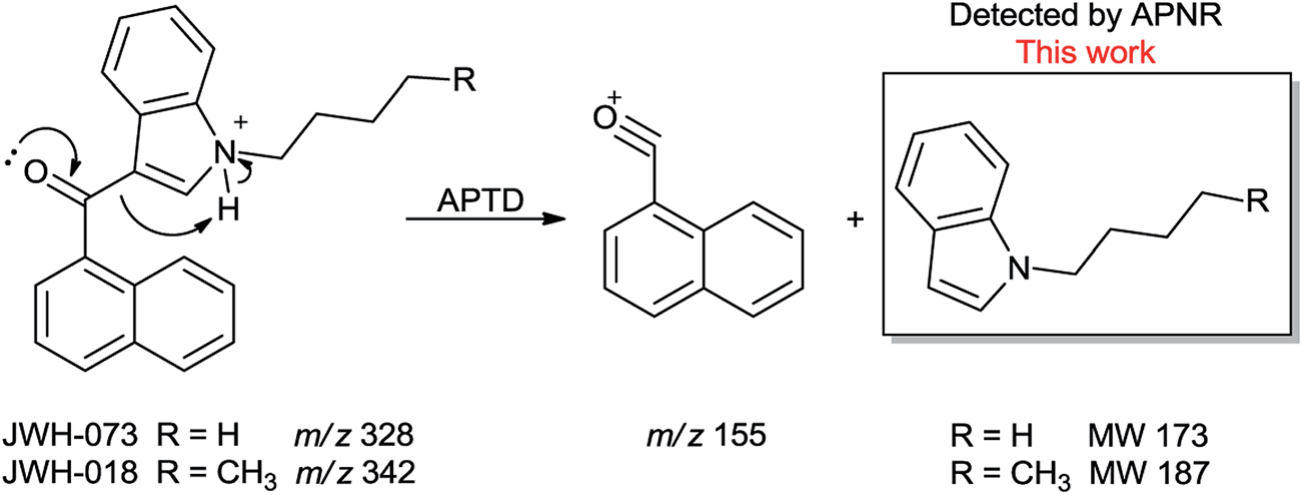
Proposed fragmentation pathway of the protonated JWH-018 and JWH-073 (ref. 38).

In addition to biomolecules, small organic compounds were also tested by APTD-MS and APNR-MS analysis. The compounds chosen in this study were two synthetic drugs naphthalen-1-yl-(1-pentylindol-3-yl)methanone (JWH-018) and naphthalen-1-yl-(1-butylindol-3-yl)methanone (JWH-073), which are naphthoylindoles containing both a naphthalene and an indole group in their structures (shown in Fig. 6). In both the CID and APTD analysis of this kind of compound, the alkylated indoles were lost during the fragmentation, leading to 1-naphthyl acylium cations. This fragmentation mechanism was proposed to involve the carbonyl C–C bond cleavage and H transfer from the initial protonated nitrogen to the cleavage carbon site (Scheme 3).^38^ As a result, the 1-naphthyl acylium cation at *m*/*z* 155 was clearly observed as in both the CID (Fig. 5Sa and Sc, ESI†) and APTD-MS (Fig. 6a and c) spectra for JWH-018 and JWH-073. The lost neutral indole moieties were completely absent from these spectra. To find evidence for the proposed fragmentation mechanism, APNR-MS analysis was applied. With the ESSI neutral reionization, the neutral generated from the fragmentation could be reionized and detected by MS. In particular, in Fig. 6b, the APNR-MS spectrum of JWH-018 showed a peak at *m*/*z* 188, corresponding to the protonated neutral fragment, 1-pentylindole (Scheme 3). Upon CID, this ion gave rise to two fragment ions at *m*/*z* 118 and 132 by cleavages of the substituted pentyl group (Fig. 5Sb, ESI†), confirming its structure. In addition, the APNR-MS spectrum of JWH-073 (Fig. 6d) displayed the reionized neutral at *m*/*z* 174 (confirmed by CID MS/MS, Fig. 5Sd, ESI†). By observing these lost neutrals, the proposed fragmentation mechanism for the formation of acylium ion and the corresponding neutral indole species from the C–C cleavage of carbonyl group is verified. The results also proved ESSI to be a soft ionization method so that the intact neutral loss fragments could be reionized and detected by MS.

All of the APTD and APNR data shown above was collected using an ion trap DECA instrument. With using a newer instrument of Orbitrap Q-Exactive Plus mass spectrometer (Thermo Fisher), we found that our APNR is quite sensitive. For instance, when the injected concentration of angiotensin II was reduced from 100 μM to 5 μM (by 20 fold), a complete series of y ions as well as b_3_, b_5_, b_6_, and b_7_ ions were still observable (Fig. 6S and Table 6S, ESI†). Furthermore, one could also reduce the sample injection flow rate. A good APNR-MS spectrum for angiotensin II was also acquired with introducing 100 μM sample at the flow rate of 0.5 μL min^–1^ instead of 10 μL min^–1^ (Fig. 7S, ESI†). To further improve the sensitivity of the system, one may need to adjust the coiled tube length, the distance between the tube outlet and the mass spectrometer instrument inlet and the position of the second sprayer.

## Conclusions

Reionization of neutrals, produced in the APTD, by the soft ionization method of ESSI makes it possible to develop a new APNR methodology for structural determination. With APNR, characteristic fragment ions could be produced by thermal dissociation, softly ionized by ESSI and detected by MS, to provide richer and/or complementary information to that gained using traditional CID-based tandem MS analysis. This study demonstrates the applicability of APNR to several types of (bio)molecules, including peptides, saccharides, nucleotides, and synthetic drugs. Results suggest that APNR, along with APTD, is versatile and sensitive, and it should have utility for both chemical structure analysis and for elucidation of ion dissociation mechanisms.
