# Supplementary material for: Atmospheric pressure neutral reionization mass spectrometry for structural analysis
Source: Chem Sci. 2017 Jul 21;8(9):6499–507. doi: 10.1039/c7sc01999h (PMC5628576; doi:10.1039/c7sc01999h)
Supplement: Supplementary file 1 [file SC-008-C7SC01999H-s001.pdf]

Electronic Supplementary Material (ESI) for Chemical Science.
This journal is © The Royal Society of Chemistry 2017

Supporting Information

Atmospheric Pressure Neutral Reionization Mass Spectrometry for Structural Analysis

Pengyuan Liu[1], Pengyi Zhao[1], R. Graham Cooks[2], and Hao Chen[1]**

[1]Center for Intelligent Chemical Instrumentation, Department of Chemistry and Biochemistry & Edison Biotechnology Institute, Ohio University, Athens, OH, USA 45701, Email: chenh2@ohio.edu

Department of Chemistry, Purdue University, 560 Oval Drive, West Lafayette, IN, USA 47907

E-mail: cooks@purdue.edu

Experimental Section

Materials Gly-His-Gly, human angiotensin II, [Arg^8]-vasopressin acetate salt, maltohexaose, maltopentaose, 3α,6α-mannopentaose, cytidine 5'-monophosphate, and adenosine 5'-diphosphate were all purchased from Sigma-Aldrich (St. Louis, MO). Glutathione (oxidized form) was purchased from TCI (Tokyo, Japan). Naphthalen-1-yl-(1-pentylindol-3-yl)methanone (denoted as JWH-018) and naphthalen-1-yl-(1-butylindol-3-yl)methanone (denoted as JWH-073) were purchased from Cayman (Ann Arbor, MI). HPLC-grade methanol and glacial acetic acid were purchased from Thermo Fisher Scientific (Waltham, MA). HPLC-grade acetonitrile was purchased from EMD Millipore (Darmstadt, Germany). Formic acid was purchased from Spectrum Chemical (New Brunswick, NJ). Deionized water used for sample preparation was obtained using a Nanopure Diamond Barnstead purification system (Barnstead International, Dubuque, IA).

Apparatus The home-made APTD device has been described before;[1] it consisted of an electrosonic spray ionization (ESSI, a variant form of electrospray ionization) sprayer[2] and a single-loop coiled stainless steel tube (3.2 mm o.d., 1.6 mm i.d., length 20 cm, loop diameter 2.5 cm) wrapped in heating tape. An LCQ Deca XP MAX (Thermo Finnigan, San Jose, CA, USA) was used to monitor the APTD-produced fragment ions. For direct APTD-MS analysis, the APTD outlet was placed ca. 1.5 cm away from the mass spectrometer inlet. The coiled tube was heated by applying 90 V to the wrapped heating tape (350°C). Sample solutions (0.1 mM) were sprayed from the first ESSI source through the coiled tube at a flow rate of 10 μL/min with the assistance of 5 kV voltage and 170 psi nebulizing N_2 gas. For APNR-MS analysis, a second ESSI sprayer was placed between the APTD device outlet and the mass spectrometer inlet to effect post-APTD ionization of the neutral species emerging from the coiled tube (Scheme 1).

For optimal sensitivity, the distance between the second sprayer and MS inlet is about 1.5 cm and the angle between the second sprayer and the coiled tube outlet is about 30-45 degree. Typically, either MeOH/H_2O/HOAc (50:50:1, v/v) or ACN/H_2O/FA (50:50:1, v/v), was used as the second ESSI spray solvent at a flow rate of 5 μL/min with the assistance of 5 kV and 170 psi nebulizing N_2 gas. In contrast to our previous report using corona discharge for neutral reionization purpose,[1] ion deflection was not applied before re-ionization in order to simplify the apparatus.

APTD and APNR-MS analysis of peptides

Table 1S. Fragment ions resulted from CID MS/MS of *b* and *y* ions generated by APNR of angiotensin II

Assigned peaks	Their fragment ions[1]
y_7 (931)	MH^+−NH_3 (914); b_6+H_2O (784); y_6 (775); b_6 (766), b_6−NH_3 (749); b_5+H_2O (687); y_5 (676); b_5 (669); b_5−NH_3 (652); a_5 (641); a_5−NH_3 (624); b_4 (532); b_4−NH_3 (515); y_4 (513); a_4 (504); a_4−NH_3 (487); b_3 (419); b_3−NH_3 (402); y_3 (400)
b_7 (881)	b_6 (784); −D−H_2O[2] (766); b_5 (647); b_5−H_2O (629); a_5 (619)
b_6 (784)	−D−H_2O (669); −D (687); b_5 (647); a_5 (619); b_4 (534); a_4 (506); b_2 (272)
y_6 (775)	y_5 (676); b_5 (610); a_5 (582); y_4 (513); b_4 (513); a_4 (485); y_3 (400); y_2 (263); b_2 (263); a_2 (235)
y_5 (676)	b_4 (511); b_3 (414); y_3 (400); y_2 (263)
b_5 (647)	b_4 (534); −D−H_2O (532); b_4−H_2O (516); −DR−H_2O (376); b_3−H_2O (353); b_2 (272); b_2−H_2O (254)
b_4 (534)	−D−H_2O (419); b_3 (371); b_3−H_2O (353); b_2 (272); b_2−H_2O (254)
y_4 (513)	y_3 (400); y_2 (263); b_2 (251); a_2 (223); y_1 (166)
y_3 (400)	y_2 (263); b_2 (235)
b_3 (371)	b_2 (272); −D−H_2O (256); b_2−H_2O (254); a_2−NH_3 (227)
b_2 (272)	−NH_3 (255); −CH_5N_3 (213)
y_2 (263)	y_1 (166)
y_1 (166)	−NH_3 (149); −CO−H_2O (120)

[1]The denotation of fragment ions listed in the 2nd column is based on the structures of their precursor ions chosen for CID MS/MS (listed in the 1st column). The same labeling rule is applied throughout the paper.

[2]Given the peptide sequence DRVYIHPF, the fragment was produced by losing the N-terminal aspartic acid, labeled (−D), from the b_7 fragment ion. The same labeling rule is applied throughout the table.

Similarly, another peptide, Gly-His-Gly (GHG), was examined. Figure 1S-a inset shows the CID MS/MS spectrum of the protonated GHG. In the spectrum, several fragment ions were observed, including *m/z* 252 from water loss, *m/z* 235 from losses of both water and ammonia, b_2 (*m/z* 195), and b_2-NH_3 (*m/z* 178). Figure 1S-a shows the APTD-MS spectrum of GHG. In the spectrum, in addition to the protonated GHG observed at *m/z* 270, some fragment ions were observed, such as *m/z* 252 from water loss, *m/z* 235 from losses of both water and ammonia, y_2 (*m/z* 213), b_2 (*m/z* 195), b_2-NH_3 (*m/z* 178), a_2 (*m/z* 167), and a_2-NH_3 (*m/z* 150), similar to those generated by CID. In contrast, the APNR-MS spectrum shown in Figure 1S-b displays more fragment ions. In addition to b_2 and y_2 ions as well as fragment ions due to losses of water and ammonia observed in the APTD-MS spectrum (as they were not removed prior to ESSI reionization), some new fragment ions were seen in Figure 1S-b. In particular, y_1 ion was observed at *m/z* 76, in combination with y_2 ion, providing adequate information to determine the sequence of this tripeptide GHG. The y_1 ion was confirmed, by observing the same fragmentation pattern with the protonated glycine in the CID MS/MS experiment which shows fragment ions *m/z* 48 by loss of CO and *m/z* 30 by consecutive losses of CO and H_2O (also in agreement with literature study[3]). The y_1 ion is likely to be produced from the ESSI-reionization of the complementary neutral that is produced during the production of b_2 ion upon APTD. In addition, side chain cleavages resulting from imidazole or methyl imidazole losses from the His residue were observed, giving rise to fragment ions at *m/z* 69 and 83. The CID MS/MS data of y_1, b_2 and y_2 ions generated by APNR is summarized in Table 2S.

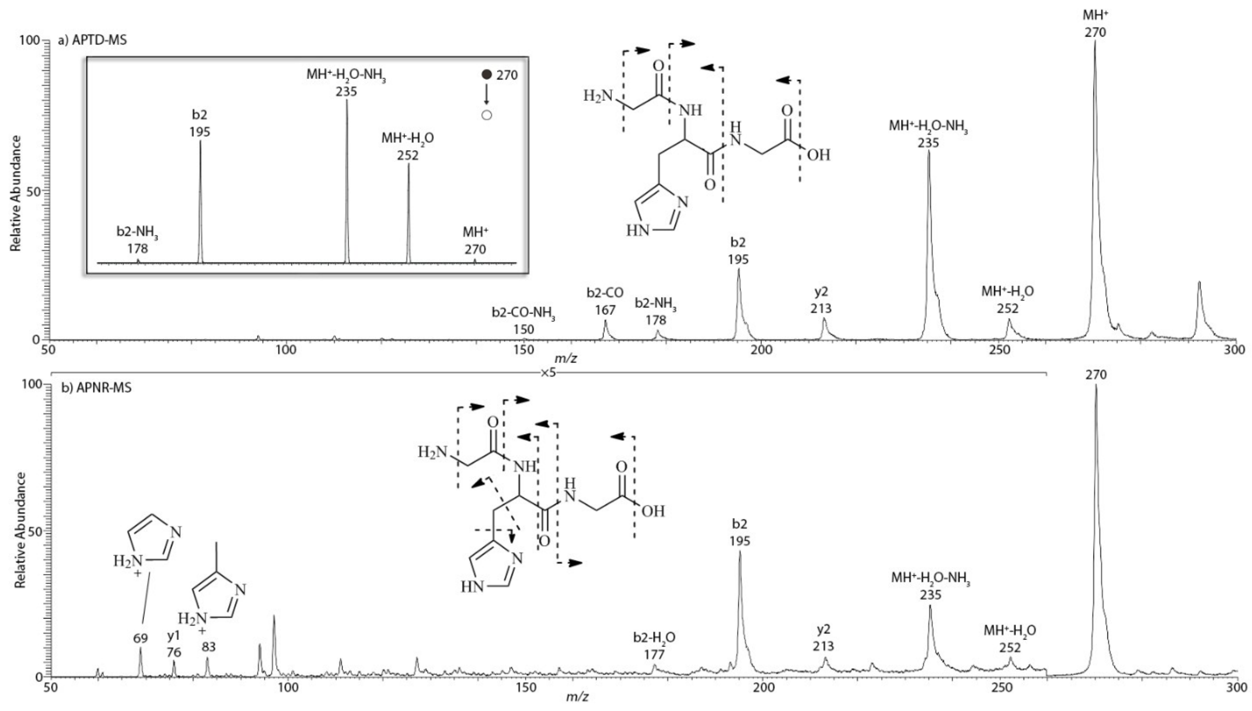

Figure 1S. a) APTD-MS and b) APNR-MS spectra of GHG.

Table 2S. Fragment ions resulted from CID MS/MS of *b* and *y* ions generated by APNR of GHG

Assigned peaks	Their fragment ions
y_2 (213)	$-H_2O$ (195); b_1 (138); a_1 (110)
b_2 (195)	$-H_2O$ (178); –CO (167); $-CH_3NO$ (150); $-C_2H_5N_2O$ (122)
y_1 (76)	–CO (48); $-CO-H_2O$ (30)

APTD and APNR-MS analysis of disulfide bond-containing peptides

Table 3S. Fragments resulted from CID MS/MS of *b* and *y* ions generated by APNR of [Arg^8]-vasopressin

Assigned peaks	Their fragment ions
M*H$^+$ (1018)	b_9* (1001); b_8* (944); y_7* (786); b_6* (691); y_6* (639); b_5* (622); y_5* (511); y_4* (397); b_3* (380); y_3 (328)
b_9* (1001)	b_5*–NH_3 (605); b_5* (622); b_3* (380); b_3*–NH_3 (363)
y_8* (949)	–NH_3 (932); b_7* (875); y_6* (639); b_5* (622); y_5* (511); y_4* (397); y_3 (328); b_2* (311)
b_5+NH_3 (673)	–2NH_3 (639); –2NH_3–H_2O (621); b_4 (542); b_4–NH_3 (525); b_3 (414); –CY[1] (390); –CY–NH_3 (373); b_2 (267); –CYF (243)
b_5 (656)	–NH_3 (639); –NH_3–H_2O (621); b_4 (542); b_4–NH_3 (525); b_3 (414); –CY (390); –CY–NH_3 (373); b_2 (267); –CYF (243)
b_5*+NH_3 (639)	–2NH_3 (605); –2NH_3 (588); b_4* (508); b_4*–NH_3 (491); b_3* (380); b_3*–NH_3 (363)
b_5* (622)	–NH_3 (605); –2NH_3 (588); b_4* (508); b_4*–NH_3 (491); b_3* (380); b_3–NH_3 (363); –CYF (243); –CYF–H_2O (225)
b_4* (508)	–NH_3 (491); b_3* (380); b_3*–NH_3 (363); –CY (276); –CY–H_2O (258)
y_4* (397)	–NH_3 (380); –2NH_3 (363); b_3* (323); b_3*–NH_3 (306); b_3*–2NH_3 (289); y_2 (231)
y_4*–NH_3 (380)	–2NH_3 (363); b_3* (323); b_3*–NH_3 (306); b_3*–2NH_3 (289)
y_3 (328)	–NH_3 (311); –NH_3–H_2O (293); b_2 (254); y_2 (231)

[1]Given the peptide sequence CYFQNCPRG, the fragment was produced by losing the N-terminal cysteine and tyrosine, labeled (−CY), from the b_5+NH_3 fragment ion. The same labeling rule is applied throughout the table.

Another intra-chain disulfide bond-containing peptide, [Arg^8]-vasotocin, was analyzed using both APTD and APNR. [Arg^8]-vasotocin has the amino acid sequence of CYIQNCPRG and a disulfide bridge linking its two cysteine residues (structure is shown in Figure 2S-a). CID MS/MS spectrum of +2 ion of [Arg^8]-vasotocin (*m/z* 526) showed no amide bond cleavage in the disulfide bond-bridged region (Figure 2S-a). In the APTD analysis of this peptide, a peak corresponding to the protonated disulfide bond elimination fragment, M*H$^+$, was observed at *m/z* 985 in the collected spectrum (Figure 2S-b); however, other fragment ions were missing. In comparison, the APNR analysis showed more fragment ions (Figure 2S-c). In details, two types

of fragment ions were observed, including those resulting from dissociation of the reduced [Arg^8]-vasotocin (e.g., b_4, b_5, y_3, and y_5) and of disulfide bond-eliminated [Arg^8]-vasotocin (e.g., M^*H^+, b_3^*, b_4^*, b_5^*, b_9^*, and y_4^*), covering most of the peptide sequence. It appears that the disulfide bond of peptide [Arg^8]-vasotocin was reduced and eliminated, upon the APTD process, as revealed by the observation of these fragment ions in APNR spectrum (Figure 2S-c). In particular, *m/z* 985 is very useful for sequencing the peptide. Upon CID, many amide bond cleavages occurred to *m/z* 985 (Figure 2S-d). All peak assignments in Figure 2S-c were confirmed by CID MS/MS (the results are summarized in Table 4S).

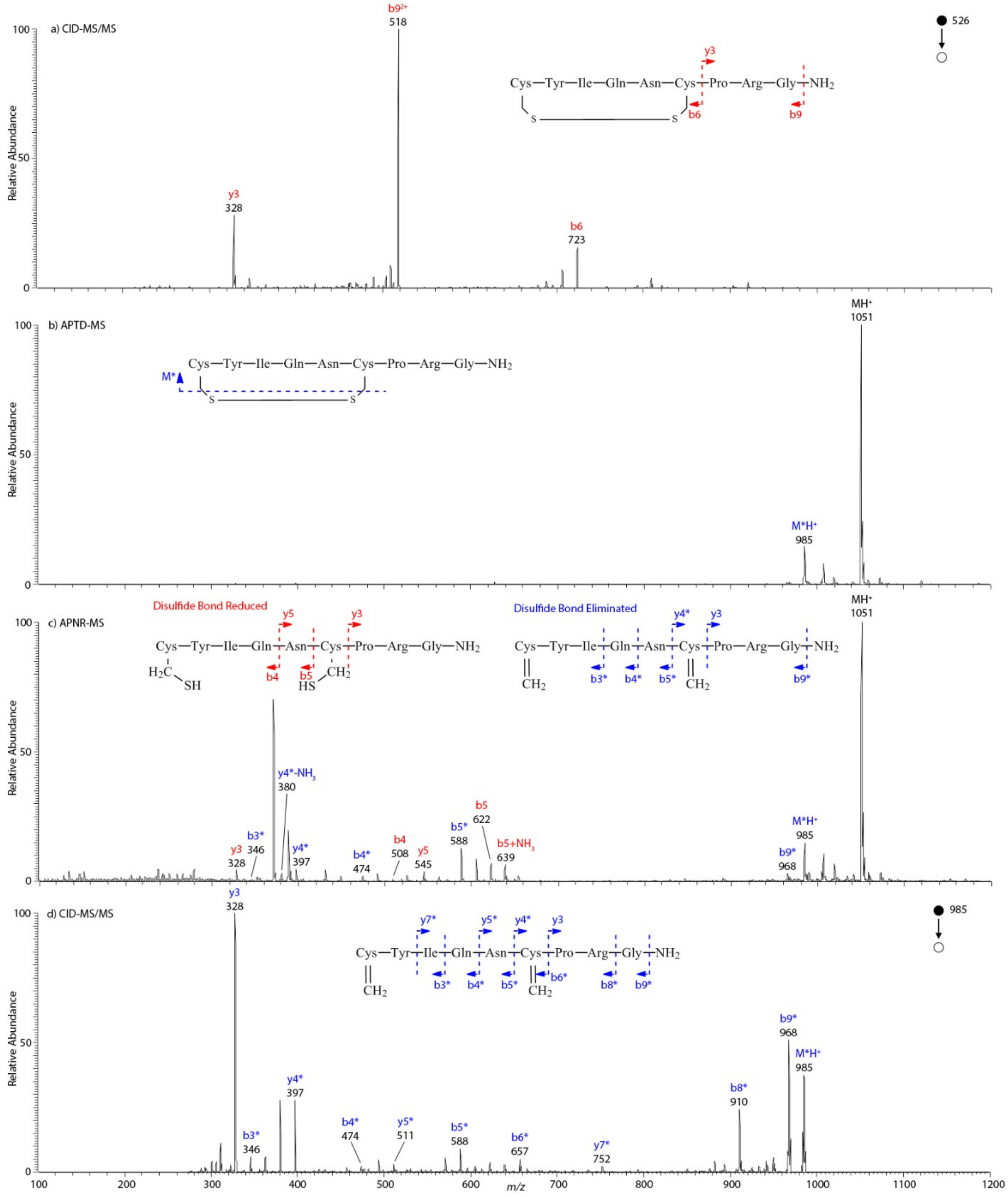

Figure 2S. a) CID MS/MS spectrum of +2 ion of [Arg^8]-vasotocin (*m/z* 526), b) APTD-MS and c) APNR-MS spectra of [Arg^8]-vasotocin; and d) CID MS/MS spectrum of M*H$^+$ at *m/z* 985.

Table 4S. Fragments resulted from CID MS/MS of *b* and *y* ions generated by APNR of [Arg^8]-vasotocin

Assigned peaks	Their fragment ions
M^*H^+ (985)	b_9* (968); b_8* (910); y_7* (752); b_6* (657); y_6* (639); b_5* (588); y_5* (511); b_4* (474); y_4* (397); b_3* (346); y_3 (328)
b_9* (968)	b_8*–NH_3 (893); b_5* (588); b_5*–NH_3 (571); b_3* (346); –CYIQN (380)
b_5+NH_3 (639)	–NH_3 (622); –2NH_3 (605); b_4 (508); b_3 (380); –CY[1] (356); b_2 (267)
b_5 (622)	–NH_3 (605); b_4 (508); b_4–NH_3 (491); b_3 (380); –CY (356); b_2 (267); –CYI (243)
b_5* (588)	–NH_3 (571); b_4* (474); b_4*–NH_3 (457); –CY (356); b_3* (346); b_3*–NH_3 (329); –CYI (243); –CYI–H_2O (225)
y_5 (545)	–NH_3 (529); y_4 (431)
b_4 (508)	–H_2O (490); b_3 (380); b_2 (267); –CY (242)
b_4* (474)	–NH_3 (457); b_3* (346); –CY (242)
y_4* (397)	–NH_3 (380); –2NH_3 (363); b_3* (323); b_3*–NH_3 (306); y_2 (231)
b_3 (380)	b_2 (267)
y_4*–NH_3 (380)	–2NH_3 (363); b_3*–NH_3 (306); b_3*–2NH_3 (289)
b_3* (346)	–NH_3 (329); b_2* (233)
y_3 (328)	–NH_3 (311); –NH_3–H_2O (293); b_2 (254); y_2 (231)

[1]Given the peptide sequence CYIQNCPRG, the fragment was produced by losing the N-terminal cysteine and tyrosine, labeled (−CY), from the b_5+NH_3 fragment ion. The same labeling rule is applied throughout the table.

Glutathione disulfide (GSSG), containing two glutathione chains linked via an inter-chain disulfide bond (structure is shown in Figure 3S-a and the two peptide chains are labeled as A Chain and B Chain, respectively), was also chosen for test. In the CID MS/MS (Figure 3S-a) and APTD-MS (Figure 3S-b) analysis of GSSG, *b* and *y* fragment ions were observed, resulting from fragmentation of either a single peptide chain or both chains. For example, y_2 ion of chain A linked with intact chain B via the disulfide bond (labeled as A(y_2)B) was observed. Also, y_2 fragments of both the chains linked via a disulfide bond (labeled as A(y_2)B(y_2)) was detected. In comparison, the APNR-MS analysis showed more fragment ions including those from peptide with reduced or eliminated disulfide bond. Since GSSG contains an inter-chain disulfide bond, while the disulfide bond elimination produces dehydroalanine from cysteine (to form chain A-

H_2S), it also leaves the other part to form chain B+S, inducing two different products. On the other hand, the reduction of the disulfide bond produces the glutathione GSH. In APNR-MS spectrum shown in Figure 3S-c, all the three products discussed above were observed as protonated species, labeled as $B+HS^+$ at *m/z* 340, $AH^+{-}H_2S$ at *m/z* 274, and AH^+ at *m/z* 308. This AH^+ ion assignment was confirmed by its CID MS/MS experiment which showed the fragment ions A(b_1) at *m/z* 130, A(y_2) at *m/z* 179, and A(b_2) at *m/z* 233 ions (Figure 3S-c inset). These fragment ions provide adequate information for determining the sequence and cysteine location of GSSG chains. Also, A(b_1) and A(y_2) ions of single chain of glutathione were observed in Figure 3S-c. Note that peptide b_1 ion is typically not stable and the observation of A(b_1) in this case is probably due to the stabilization of the particular A(b_1) ion structure by the carboxyl group of *r*-glutamic acid residue.[4] The assignments in the APNR spectrum were confirmed by CID MS/MS experiments (results are summarized in Table 5S).

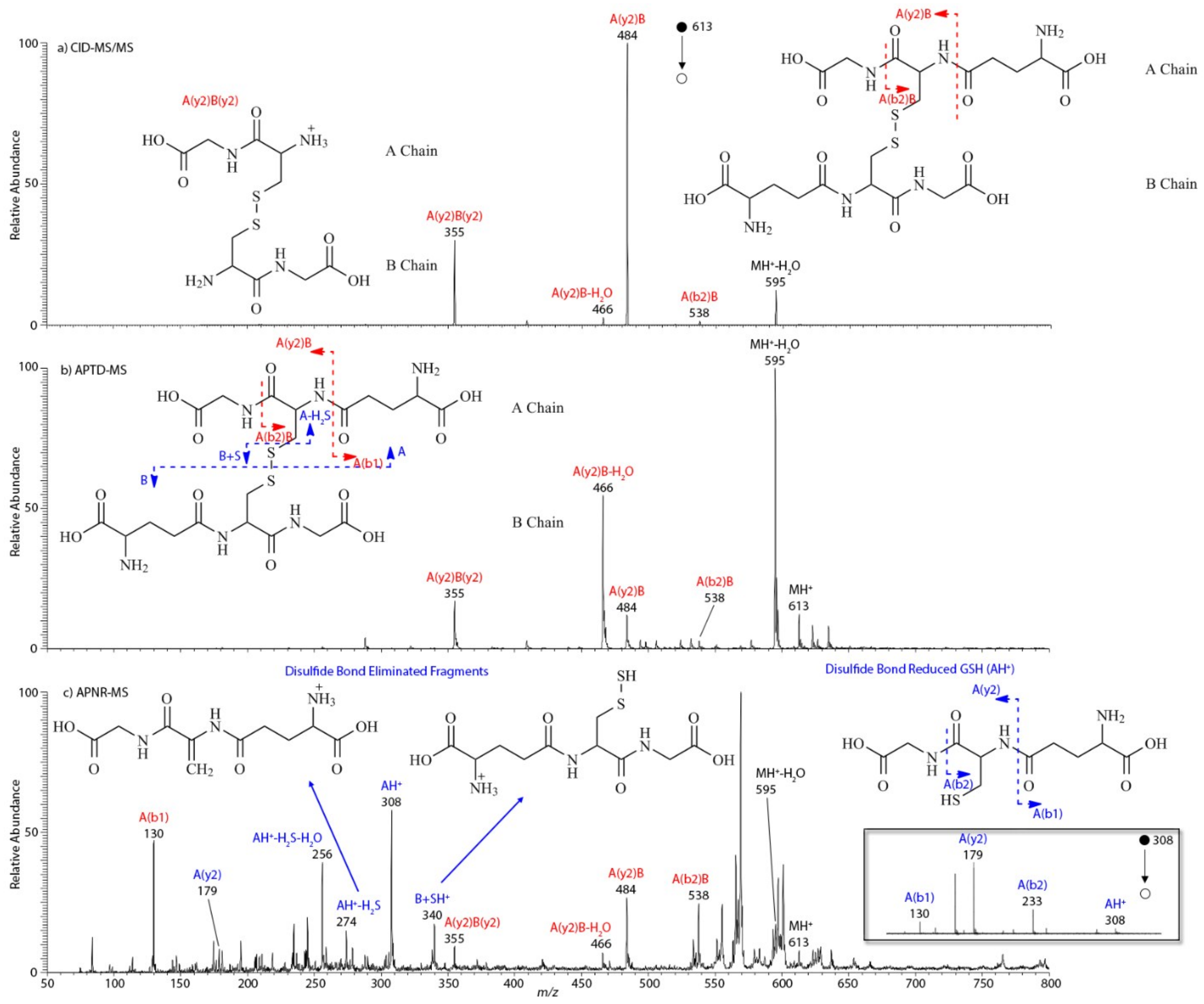

Figure 3S. a) CID MS/MS of +1 ion of GSSG (*m/z* 613), b) APTD-MS and c) APNR-MS spectra of GSSG

Table 5S. Fragments resulted from CID MS/MS of *b* and *y* ions generated by APNR of GSSG

Assigned peaks	Their fragment ions
MH–H_2O (595)	–H_2O (577); A(b_2)B–H_2O (520); A(y_2)B–H_2O (466); A(b_2)B(y_2)–H_2O (391); A+HS^+ (340); A+HS^+–H_2O (322); A–H_2O–H_2 (288); AH^+–H_2S–H_2O (256)
A(b_2)B (538)	A(b_2)B(b_2) (464); A(b_2)B(y_2) (409); A(b_2) (231)
A(y_2)B (484)	–H_2O (466); A(b_2)B(y_2) (409); A(y_2)B(y_2) (355)
A(y_2)B–H_2O (466)	A(y_2)B(b_2)–H_2O (391); A(y_2)B(y_2)–H_2O (337); B–H_2O–H_2 (288); B–H_2O–H_2S (256); B(b_2)–H_2O–H_2S (181)
A(y_2)B(y_2) (355)	–H_2O (337); –CH_3NO_2 (294); A(y_2)+S (211); A(y_2) (179); A(y_2)–CH_4S (131)

B+SH$^+$ (340)	–H_2O (322); –2H_2O (304); A(b_2)+S (265); A(y_2)+S (211); A(y_2)+S–H_2S (177); A(y_2)–S (147); A(b_1) (130)
AH$^+$ (308)	–H_2O (290); A(b_2) (233); A(b_2)–H_2O (215); A(y_2) (179); A(y_2)–NH_3 (162)
AH$^+$–H_2S (274)	–H_2O (256); A(b_2)–H_2S (199); A(y_2)–H_2S (145); A(b_1) (130); A(y_2)–H_2S–H_2O (127); A(y_2)–H_2S–HCOOH (99)
AH$^+$–H_2S–H_2O (256)	A(b_2)–H_2O–H_2S (181)
A(y_2) (179)	–NH_3 (162); –H_2S-H (144); y_1 (76)
A(b_1) (130)	–H_2O (112); –CO (102); –CO–H_2O (84)

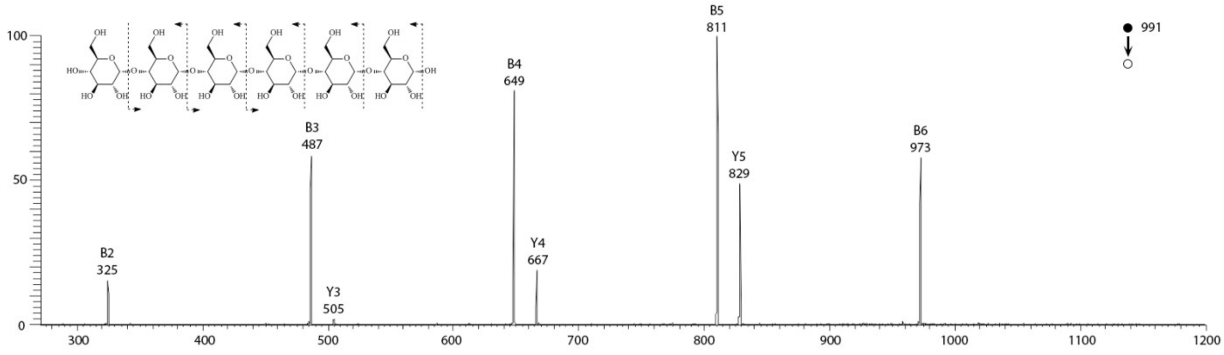

Figure 4S. CID MS/MS spectrum of the protonated maltohexaose (m/z 991)

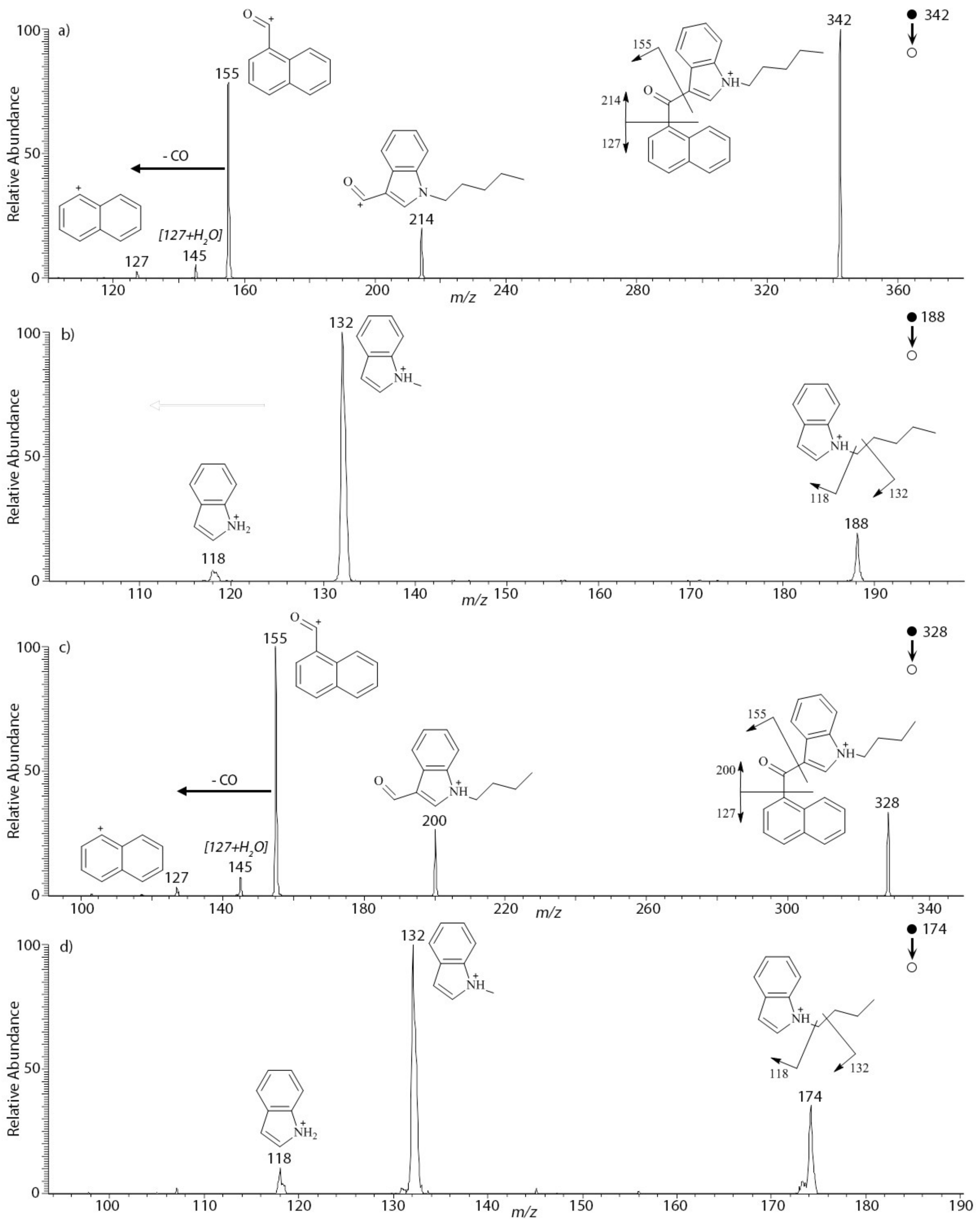

Figure 5S. CID MS/MS spectra of a) the protonated JWH-018 (*m/z* 342) and c) the protonated JWH-073 (*m/z* 328); and the CID MS/MS spectra of reionized indoles resulting from the APTD of b) JWH-018 (*m/z* 188) and d) JWH-073 (*m/z* 174).

Sensitivity evaluation of APNR

The sensitivity of APNR was tested with angiotensin II as a model compound using an Orbitrap Q-Exactive Plus mass spectrometer. In the test, different concentrations of angiotensin II solutions were prepared for APNR experiment, including 100 µM, 50 µM, 20 µM, 10 µM, 5 µM, 2 µM, and 1 µM. The sample solutions were infused at a constant flow rate of 10 µL/min across the test. For each individual test, the fragment ions were monitored and listed in Table 6S. As shown in the table, a complete series of y ions as well as b_3, b_5, b_6, and b_7 ions were observed when the sample concentration was 5 µM (Figure 6S) or higher. When the sample concentration was reduced to 2 µM, most APNR fragments were still observed; however, no fragment ion was observed when the sample concentration was further reduced to 1 µM. In conclusion, APNR could provide fairly good sensitivity and peptide sample with concentration as low as 5 µM could be analyzed by APNR.

Table 6S. Sensitivity test on APNR experiment of angiotensin II

Fragment ions	Sample centration (µM)						
	100	50	20	10	5	2	1
y_7 (931)	D[1]	D	D	D	D	D	n.d.[2]
b_7 (881)	D	D	D	D	D	n.d.	n.d.
b_6 (784)	D	D	D	D	D	D	n.d.
y_6 (775)	D	D	D	D	D	n.d.	n.d.
y_5 (676)	D	D	D	D	D	D	n.d.
b_5 (647)	D	D	D	D	D	D	n.d.
y_4 (513)	D	D	D	D	D	n.d.	n.d.
y_3 (400)	D	D	D	D	D	D	n.d.
b_3 (371)	D	D	D	D	D	n.d.	n.d.
y_2 (263)	D	D	D	D	D	D	n.d.
y_1 (166)	D	D	D	D	D	D	n.d.

[1]The capital letter "D" here represents "detected". The same rule is applied throughout the paper.
[2]The letter "n.d." here represents "not detected". The same rule is applied throughout the paper.

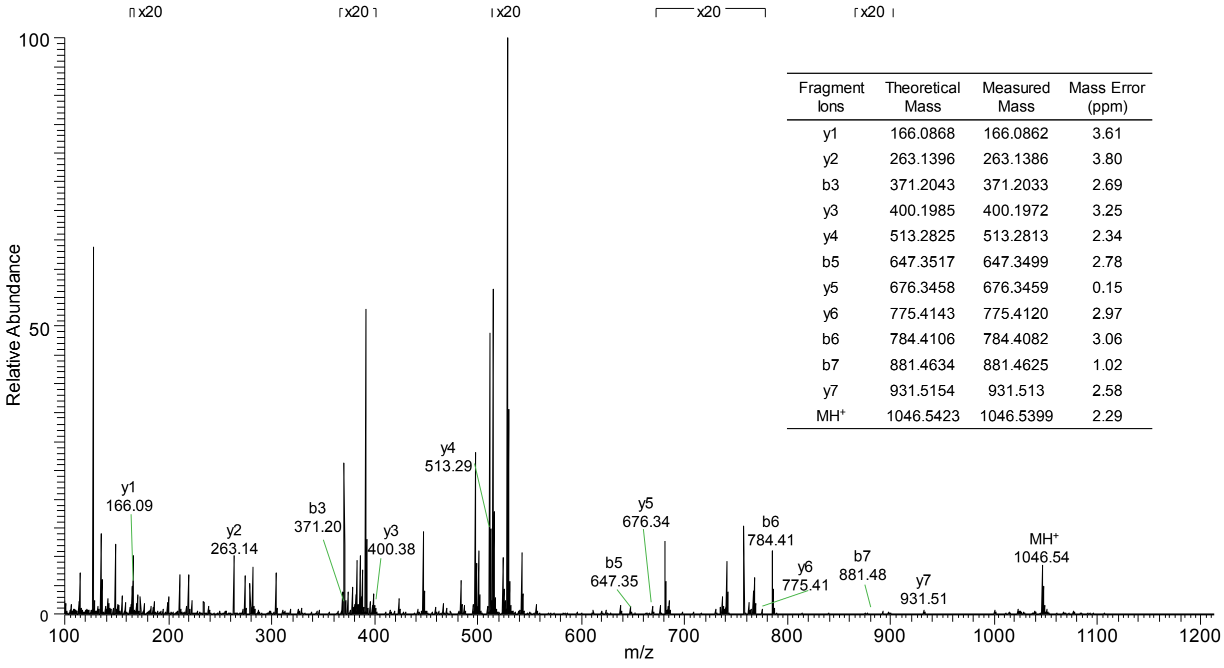

Fragment Ions	Theoretical Mass	Measured Mass	Mass Error (ppm)
y1	166.0868	166.0862	3.61
y2	263.1396	263.1386	3.80
b3	371.2043	371.2033	2.69
y3	400.1985	400.1972	3.25
y4	513.2825	513.2813	2.34
b5	647.3517	647.3499	2.78
y5	676.3458	676.3459	0.15
y6	775.4143	775.4120	2.97
b6	784.4106	784.4082	3.06
b7	881.4634	881.4625	1.02
y7	931.5154	931.513	2.58
MH^+	1046.5423	1046.5399	2.29

Figure 6S. APNR-MS spectrum of 5 µM angiotensin II with the injection flow rate of 10 µL/min

The sample injection flow rate for APNR was also tested with angiotensin II as a model compound using an Orbitrap Q-Exactive Plus mass spectrometer. In the test, different sample injection flow rates were tested, including 10 µL/min, 5 µL/min, 2 µL/min, 1 µL/min, and 0.5 µL/min. For each individual test, the fragment ions were monitored and listed in Table 7S. As shown in the table, a complete series of *y* ions as well as b_3, b_5, b_6, and b_7 ions were observed across the entire test, even when the flow rate was reduced to 0.5 µL/min (spectrum is shown in Figure 7S). The result suggested that APNR technique tolerates a low sample injection flow rate.

Table 7S. Flow rate test on APNR experiment of angiotensin II

Fragment ions	Flow rate (µL/min)				
	10	5	2	1	0.5
y_7 (931)	D	D	D	D	D
b_7 (881)	D	D	D	D	D
b_6 (784)	D	D	D	D	D

y_6 (775)	D	D	D	D	D
y_5 (676)	D	D	D	D	D
b_5 (647)	D	D	D	D	D
y_4 (513)	D	D	D	D	D
y_3 (400)	D	D	D	D	D
b_3 (371)	D	D	D	D	D
y_2 (263)	D	D	D	D	D
y_1 (166)	D	D	D	D	D

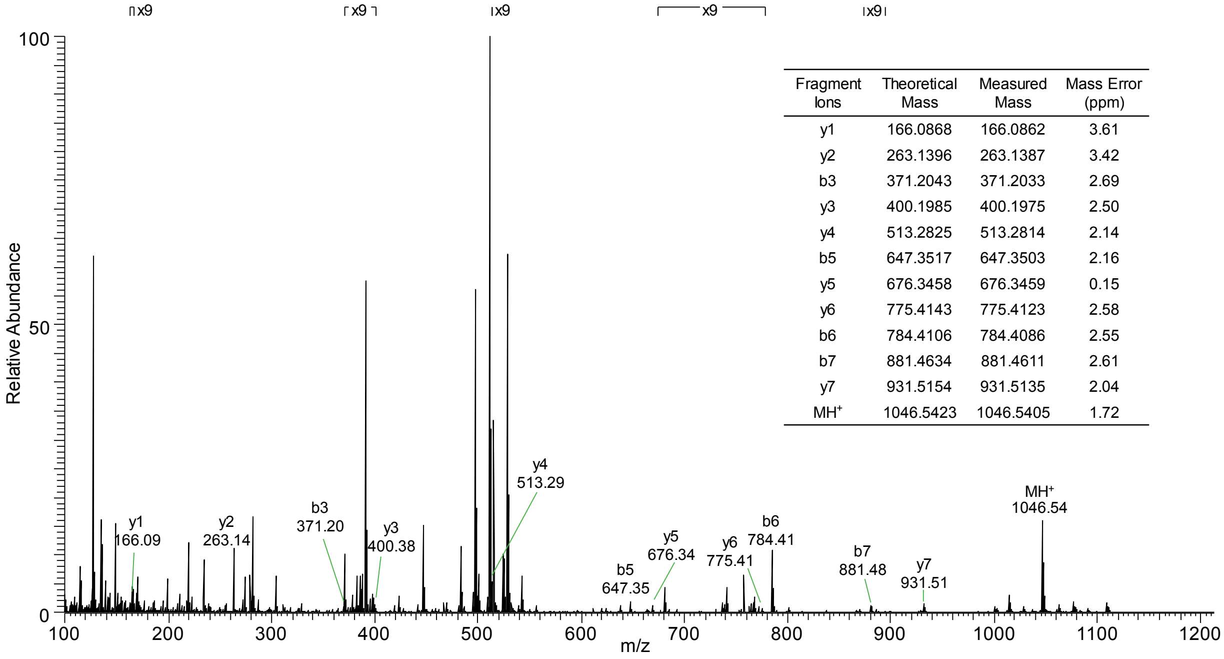

Figure 7S. APNR-MS spectrum of 100 μM angiotensin II with the injection flow rate of 0.5 μL/min

References:

[1] H. Chen, L. S. Eberlin, R. G. Cooks, *J. Am. Chem. Soc.* **2007**, *129*, 5880-5886.

[2] Z. Takáts, J. M. Wiseman, B. Gologan, R. G. Cooks, *Anal. Chem.* **2004**, *76*, 4050-4058.

[3] R. A. J. O'Hair, P. S. Broughton, M. L. Styles, B. T. Frink, C. M. Hadad, *J. Am. Soc. Mass Spectrom.* **2000**, *11*, 687-696.

[4] [a]R. A. J. O'Hair, G. E. Reid, *Rapid Commun. Mass Spec.* **2000**, *14*, 1220-1225; [b]R. Ramanathan, K. Cao, E. Cavalieri, M. L. Gross, *J. Am. Soc. Mass Spectrom.* **1998**, *9*, 612-619.
